# Primary Care Follow-Up After Mental Health and Substance Use Emergency Department Visits in Medicaid

**DOI:** 10.1001/jamanetworkopen.2026.4917

**Published:** 2026-04-14

**Authors:** Jonathan A. Staloff, Edwin S. Wong, Joseph H. Joo, Joshua M. Liao, Christopher P. Chen, Judy Zerzan-Thul, Lingmei Zhou, Ashok Reddy

**Affiliations:** 1 Department of Family Medicine, University of Washington, Seattle; 2 Department of Health Systems and Population Health, University of Washington School of Public Health, Seattle; 3 Department of Internal Medicine, University of Texas Southwestern Medical Center, Dallas; 4 Department of Medicine, University of Washington, Seattle; 5 Washington Health Care Authority, Olympia

## Abstract

**Importance:**

Medicaid beneficiaries—particularly those with mental health (MH) conditions, substance use disorder (SUD), and alcohol use disorder (AUD)—frequently use emergency departments (EDs). Timely primary care follow-up can reduce high 30-day ED revisit rates for these conditions; however, primary care follow-up after such ED visits remains understudied.

**Objective:**

To examine rates of 30-day condition-concordant primary care follow-up for MH, SUD, and AUD after ED visits related to these conditions and identify characteristics associated with condition-concordant primary care follow-up.

**Design, Setting, and Participants:**

This cohort study retrospectively analyzed 2022 Medicaid claims data from beneficiaries residing in Washington State who had at least 1 ED visit during the study period. Data were analyzed between March and May 2025.

**Exposure:**

ED visits for MH conditions, SUDs, or AUD.

**Main Outcomes and Measures:**

The primary outcome was primary care follow-up for MH conditions or SUDs within 30 days following ED visits for those diagnoses (condition-concordant primary care follow-up), and the secondary outcome was condition-concordant primary care follow-up for AUD within the same time frame. Multivariable logistic regression with marginal effects was used to assess associations between beneficiary characteristics and probability of primary care follow-up.

**Results:**

This study included 859 043 Medicaid ED visit claims from 367 245 unique individuals in Washington (mean [SD] age, 41.7 [16.2] years); 496 775 claims (57.8%) were for women. Among the claims, 52 500 (6.1%) were for beneficiaries self-reporting as Alaska Native or American Indian, 48 103 (5.6%) as Asian or Pacific Islander, 89 548 (10.4%) as non-Hispanic Black, 476 968 (55.5%) as non-Hispanic White; and 173 996 (20.3%) as other race (17 928 [2.1%] did not provide or had missing information on race); 142 556 (16.6%) claims were for beneficiaries self-reporting as Hispanic and 678 961 (79.0%) as non-Hispanic (37 526 [4.4%] did not provide or had missing information on ethnicity). Condition-concordant primary care follow-up occurred after 18 722 of 131 704 MH condition– (14.2%), 11 353 of 101 684 SUD (11.2%)-, and 3675 of 33 196 AUD (11.1%)-related ED visits. All racial groups, and non-Hispanic White individuals in particular, had a higher probability of receiving condition-concordant primary care follow-up than non-Hispanic Black individuals (estimated marginal effects: MH, 4.47 [95% CI, 3.87-5.07] percentage points; SUD, 4.70 [95% CI, 4.12-5.27] percentage points; AUD, 4.00 [95% CI, 2.87-5.13] percentage points). Individuals experiencing homelessness had a significantly lower probability of receiving condition-concordant primary care follow-up (estimated marginal effects: MH, −2.74 [95% CI, −3.25 to −2.23] percentage points; SUD, −1.88 [95% CI, −2.33 to −1.44] percentage points; AUD, −1.86 [95% CI, −2.73 to −0.99] percentage points) compared with those not experiencing homelessness.

**Conclusions and Relevance:**

In this cohort study of Medicaid beneficiaries in Washington, condition-concordant primary care follow-up after ED visits for MH conditions, SUDs, and AUD was infrequent. Observed racial and social differences suggest potential barriers to care access and coordination following ED visits. Tailored care coordination and outreach may be necessary to improve continuity of and access to primary care services among these populations.

## Key Points

**Question:**

What are the rates of and characteristics associated with primary care follow-up after emergency department (ED) visits for mental health (MH) conditions, substance use disorders (SUDs), and alcohol use disorder (AUD) among Washington State Medicaid beneficiaries?

**Findings:**

In this cohort study of 859 043 Medicaid ED visit claims for 367 245 beneficiaries in Washington, fewer than 15% had condition-concordant primary care follow-up after ED visits for MH conditions, SUDs, or AUD. Rates were markedly lower among non-Hispanic Black individuals and people experiencing homelessness.

**Meaning:**

These findings suggest racial and social differences in care access and coordination after ED visits, indicating a potential need for tailored care coordination and outreach in some populations.

## Teaser

This cohort study examines the probability of receiving condition-concordant primary care follow-up after emergency department visits for mental health conditions or substance use disorders among Medicaid beneficiaries in Washington State.

## Introduction

Medicaid beneficiaries frequently use emergency departments (EDs) and have high 30-day ED revisit rates.^1,2,3^ Living with mental health (MH) conditions or substance use disorders (SUDs), including alcohol use disorder (AUD), is among the factors strongly associated with frequent ED use, and US Medicaid is the primary payer for a large proportion of ED visits for these conditions.^4,5,6,7,8,9^ Indeed, 30-day ED revisit rates have been found to be approximately 25% among Medicaid beneficiaries and as high as 40% for individuals with SUD- and AUD-related ED visits.^1,10^ Prior studies have demonstrated that primary care follow-up, often associated with multidisciplinary behavioral health programs, can reduce recurrent ED use and hospitalizations.^11^ Primary care follow-up is essential because it provides the sustained, comprehensive care necessary to manage MH conditions, SUDs, and AUD and support long-term recovery.

However, insight into primary care follow-up after ED visits for MH conditions, SUDs, and AUD remains limited. Prior work has demonstrated that approximately 37% of Medicaid beneficiaries completed any type of ambulatory follow-up visit within 30 days of an ED visit for any condition or reason.^1^ Earlier research also suggests that Medicaid beneficiaries who visited the ED for an MH condition or SUD were less likely to receive any type of ambulatory follow-up within 7 or 30 days compared with beneficiaries who visited the ED for other reasons.^12^ In contrast with these general findings, data about primary care follow-up after ED visits for MH conditions, SUDs, and AUD are lacking. Furthermore, no contemporary studies have evaluated characteristics associated with condition-concordant primary care follow-up after such ED visits.

Determining whether Medicaid beneficiaries with MH conditions, SUDs, or AUD receive timely primary care follow-up after ED visits is essential for designing effective interventions to reduce ED use, improve continuity of care, and address differences in behavioral health outcomes. By addressing this evidence gap, our study can provide timely insight for health systems and policymakers working to strengthen the role of primary care in behavioral health. Through its Medicaid program, Washington State has taken significant steps to expand access to behavioral health care and support better integration between behavioral and physical health services, making it an ideal setting for this study. Beginning in 2016, Washington launched an integrated managed care initiative, which required that Washington Medicaid managed care organizations be responsible for improving the integration and coordination of both behavioral and physical health services as part of their health plan products, with full statewide implementation by 2020. This reform aimed to improve care coordination and integrate primary care and behavioral health services in the Medicaid program.^13^ Therefore, this study aimed to examine rates of 30-day primary care follow-up for MH conditions, SUDs, or AUD after ED visits related to these conditions and identify characteristics associated with the probability of primary care follow-up among Medicaid beneficiaries living in Washington.

## Methods

This retrospective cohort study used 2022 Medicaid claims data and was approved by the institutional review board of the University of Washington, which waived informed consent because data were deidentified. We followed Strengthening the Reporting of Observational Studies in Epidemiology (STROBE) reporting guidelines for cohort studies.^14^

We included claims for individuals aged 18 or older who resided in Washington, were enrolled in Medicaid for at least 11 months of the year, and had at least 1 ED visit during the study period. The primary exposure variable was condition-specific ED visits, which we categorized as being for MH conditions or for all SUDs. ED visits were identified using outpatient claim *Current Procedural Terminology* codes 99281 to 99285, inpatient claim revenue codes 0450 to 0459 and 0981, and place of service code 23. ED visit claims that overlapped with outpatient claims within 1 day were removed from the sample. We used *International Statistical Classification of Diseases and Related Health Problems, Tenth Revision* diagnosis codes to identify MH conditions (eMethods 1 in Supplement 1) and SUDs (eMethods 2 in Supplement 1), which were organized in code groupings used in prior published research.^15^ We also conducted a subgroup analysis of primary care follow-up for AUD as a specific type of SUD because of recent Washington initiatives to systematically integrate prevention and treatment services for AUD into primary care.^16^

The 2 primary outcome variables were primary care follow-up for MH conditions or any SUD within 30 days of an MH condition– or SUD-related ED visit, respectively (30-day condition-concordant primary care follow-up). The secondary outcome variable was 30-day condition-concordant primary care follow-up after an AUD-related ED visit. For each outcome, consistent with prior work, telehealth and in-person primary care follow-up visits were identified using *Current Procedural Terminology* codes (eMethods 3 in Supplement 1).^1^ Using previously validated methods, follow-up visits were defined as primary care follow-up if both the clinician providing the service (eg, family medicine physician) and billing entity (eg, single specialty group practice) were each consistent with primary care delivery.^17^ Based on this approach, we considered an individual to have received 30-day condition-concordant primary care follow-up if claims for the index ED visit and primary care follow-up visit had the same primary diagnosis code.

### Statistical Analysis

In our analysis, we first described the proportion of 30-day condition-concordant primary care follow-up for each subset of ED visits for either MH conditions, SUDs, or AUD. Next, using complete case analyses, we used multivariable logistic regression to identify beneficiary characteristics associated with higher or lower probability of receiving 30-day condition-concordant primary care follow-up. Missingness was less than 10% in all models, and thus, we used a complete case analysis approach.^18,19^ Separate models were used for 30-day condition-concordant primary care follow-up for MH conditions and SUDs and for AUD in the subgroup analysis. All models included beneficiary age, sex, race, ethnicity, residence area (rural area resident or not rural area resident based on Rural Urban Commuting Area Score),^20^ individual federal poverty level, homelessness status (defined as experiencing homelessness for at least 1 month during the last 12 months; self-reported at the time of Medicaid enrollment), primary speaking language (self-reported), and modified Quan-Charlson Comorbidity Index (QCCI) score. Race and ethnicity data were self-reported by Medicaid beneficiaries at the time of enrollment. Initial self-report options were Alaska Native, American Indian, Asian, Native Hawaiian or Other Pacific Islander, non-Hispanic Black, non-Hispanic White, not provided or missing, and other race. Due to limited sample sizes for some racial groups, race was subsequently categorized as Alaska Native or American Indian, Asian or Pacific Islander, non-Hispanic Black, non-Hispanic White, other race, or not provided or missing. The other race group included those who identified as Hawaiian or other race at the time of enrollment. Ethnicity was categorized as Hispanic, not Hispanic, or not provided or missing, also via self-report. Race and ethnicity were included as covariates because racial and ethnic differences in health care access and use can confound associations between ED visits and subsequent primary care follow-up, and adjustment helps account for systematic differences in health care access.^21^

Collectively, these variables were chosen a priori to reflect the clinical and social factors that can contribute to health care use patterns and that, if unadjusted for, could potentially confound findings. To improve interpretability, estimated marginal effects (MEs) reflecting differences in probability of condition-concordant follow-up were used to report model results. In all analyses, statistical significance was assessed using 2-sided tests with an α level of 0.05. This analysis was conducted between March and May 2025 using SAS 9.2 (SAS Institute).

## Results 

Among the 367 245 Medicaid beneficiaries in Washington who had 859 043 ED visit claims, the mean (SD) age at the time of the ED claim was 41.7 (16.2) years; 496 775 claims (57.8%) were for women, and 350 789 (40.8%) were for men. Among the claims, 52 500 (6.1%) were for beneficiaries self-reporting as Alaska Native or American Indian; 48 103 (5.6%), Asian or Pacific Islander; 89 548 (10.4%), non-Hispanic Black; 476 968 (55.5%), non-Hispanic White; and 173 996 (20.3%), other race (17 928 [2.1%] did not provide or had missing information on race). A total of 142 556 (16.6%) claims were for beneficiaries self-reporting as Hispanic and 678 961 (79.0%) as non-Hispanic (37 526 [4.4%] did not provide or had missing information on ethnicity). Homelessness was experienced by beneficiaries in 12.6% of the claims, 93.3% were for primarily English-speaking beneficiaries, and 97.6% involved nonrural residents; the mean (SD) modified QCCI score was 1.26 (SD 2.40) (Table 1). The mean (SD) number of ED visits in this population was 1.94 (2.79) for those with MH condition–, 1.96 (2.66) for those with SUD-, and 1.98 (2.95) for those with AUD-related ED visits. Among 131 704 MH condition–related ED visits, 18 722 (14.2%) had 30-day condition-concordant primary care follow-up. Among 101 684 SUD-related ED visits, 11 353 (11.2%) had condition-concordant primary care follow-up. In our subgroup analysis, among 33 196 claims for AUD-related ED visits, 3675 (11.1%) had condition-concordant primary care follow-up.

**Table 1.** Characteristics of Beneficiaries in Cohort

Characteristic	Claims for ED visits, No. (%)			
	All (N = 859 043)	Condition related		
		Mental health condition (n = 131 704)	Substance use disorder (n = 101 684)	Alcohol use disorder (n = 33 196)
**Demographic characteristics**				
Age, mean (SD), y	41.7 (16.2)	40.7 (14.6)	40.7 (13.0)	43.6 (13.3)
Sex				
Male	350 789 (40.8)	55 129 (41.9)	60 031 (59.0)	20 637 (62.2)
Female	496 775 (57.8)	75 295 (57.2)	40 455 (39.8)	12 021 (36.2)
Missing	11 479 (1.3)	1280 (1.0)	1198 (1.2)	538 (1.6)
Race^a^				
Alaska Native or American Indian	52 500 (6.1)	7980 (6.1)	9565 (9.4)	3743 (11.3)
Asian or Pacific Islander	48 103 (5.6)	4144 (3.2)	2808 (2.8)	857 (2.6)
Non-Hispanic Black	89 548 (10.4)	12 706 (9.7)	10 327 (10.2)	2726 (8.2)
Non-Hispanic White	476 968 (55.5)	84 121 (63.9)	62 659 (61.6)	20 211 (60.9)
Other	173 996 (20.3)	20 369 (15.5)	15 050 (14.8)	5008 (15.1)
Not provided or missing	17 928 (2.1)	2384 (1.8)	1275 (1.3)	651 (2.0)
Ethnicity				
Hispanic	142 556 (16.6)	15 667 (11.9)	12 417 (12.2)	3943 (11.9)
Not Hispanic	678 961 (79.0)	110 828 (84.2)	85 933 (84.5)	27 628 (83.2)
Not provided or missing	37 526 (4.4)	5209 (4.00)	3334 (3.3)	1625 (4.9)
Residence area				
Nonrural	838 243 (97.6)	128 775 (97.8)	99 115 (97.5)	32 243 (97.1)
Rural	20 800 (2.4)	2929 (2.2)	2569 (2.5)	953 (2.9)
Individual federal poverty level, mean (SD), %	31.5 (50.8)	25.5 (47.0)	19 (41.2)	23.2 (45.4)
Experiencing homelessness	110 425 (12.9)	20 345 (15.5)	25 095 (24.7)	6180 (18.6)
Primary speaking language				
Not English	44 977 (5.2)	2617 (2.0)	1350 (1.3)	721 (2.2)
English	801 135 (93.3)	127 004 (96.4)	99 392 (97.8)	32 223 (97.1)
Missing	12 931 (1.5)	2083 (1.6)	942 (0.9)	252 (0.8)
Modified QCCI score, mean (SD)	1.26 (2.40)	1.33 (2.43)	1.42 (2.61)	1.55 (2.61)
**Presence of common comorbid conditions**				
Acute myocardial infarction	21 344 (2.5)	3129 (2.4)	3233 (3.2)	1000 (3.0)
Congestive heart failure	56 422 (6.6)	7237 (5.5)	7405 (7.3)	2081 (6.3)
Peripheral vascular disease	21 522 (2.5)	2503 (1.9)	2100 (2.1)	658 (2.0)
Cerebrovascular disease	42 968 (5.0)	7396 (5.6)	5729 (5.6)	2493 (7.5)
Chronic pulmonary disease	109 195 (12.7)	19 830 (15.1)	10 882 (10.7)	3358 (10.1)
Diabetes without chronic complications	106 294 (12.4)	15 796 (12.0)	8087 (8.0)	2620 (7.9)
Diabetes with chronic complications	52 251 (6.1)	6729 (5.1)	3266 (3.2)	911 (2.7)
Cancer	23 066 (2.7)	2713 (2.1)	1513 (1.5)	461 (1.4)
HIV/AIDS	60 118 (7.0)	11 529 (8.8)	10 289 (10.1)	3794 (11.4)

Abbreviations: ED, emergency department; QCCI, Quan-Charlson Comorbidity Index.

^a^ Due to small sample sizes, Alaska Native and American Indian were initially separate racial identity categories that were consolidated into 1 category, as were Asian and Pacific Islander. Other race included those who identified as Hawaiian or other race at the time of enrollment.

### Thirty-Day Condition-Concordant Primary Care Follow-Up After MH Condition–Related ED Visits

Among Medicaid beneficiaries with ED visits for MH conditions, characteristics associated with higher marginal probability of receiving 30-day condition-concordant primary care follow-up included female sex (estimated ME, 2.49 [95% CI, 2.09-2.89] percentage points; referent, male sex); Alaska Native or American Indian (estimated ME, 2.03 [95% CI, 1.10-2.96] percentage points), Asian or Pacific Islander (estimated ME, 4.00 [95% CI, 2.76-5.24] percentage points), non-Hispanic White (estimated ME, 4.47 [95% CI, 3.87-5.07] percentage points), or other race (estimated ME, 4.07 [95% CI, 2.83-5.30] percentage points) (referent, non-Hispanic Black); rural residence (estimated ME, 4.73 [95% CI, 3.26-6.22] percentage points; referent, urban); and higher modified QCCI score (estimated ME, 0.37 [95% CI, 0.29-0.47] percentage points). Characteristics associated with lower marginal probability of receiving 30-day condition-concordant primary care follow-up after an ED visit for MH conditions included older age (estimated ME, −0.11 [95% CI, −0.12 to −0.09] percentage points) and experiencing homelessness (estimated ME, −2.74 [95% CI, −3.25 to −2.23] percentage points; referent, not experiencing homelessness) (Table 2).

**Table 2.** Estimated Marginal Effects of Characteristics Associated With 30-Day Primary Care Follow-Up Visit for MH Conditions, SUDs, and AUD^a^

Characteristic	Estimated Marginal effect (95% CI), percentage points		
	Model 1: mental health (n = 121 392)^b^	Model 2: SUD (n = 95 441)^c^	Model 3: AUD (n = 30 527)^d^
Age	−0.11 (−0.12 to −0.09)	0.05 (0.04 to 0.07)	0.04 (0.01 to 0.07)
Sex			
Female	2.49 (2.09 to 2.89)	0.27 (−0.14 to 0.68)	0.96 (0.21 to 1.70)
Male	0 [Reference]	0 [Reference]	0 [Reference]
Race^e^			
Alaska Native or American Indian	2.03 (1.10 to 2.96)	3.51 (2.68 to 4.33)	2.51 (1.05 to 3.96)
Asian or Pacific Islander	4.00 (2.76 to 5.24)	3.06 (1.79 to 4.33)	2.47 (0.08 to 4.86)
Non-Hispanic Black	0 [Reference]	0 [Reference]	0 [Reference]
Non-Hispanic White	4.47 (3.87 to 5.07)	4.70 (4.12 to 5.27)	4.00 (2.87 to 5.13)
Other	4.07 (2.83 to 5.30)	3.47 (2.21 to 4.71)	3.07 (0.72 to 5.42)
Hispanic	0.61 (−0.58 to 1.80)	0.62 (−0.65 to 1.89)	−0.07 (−2.29 to 2.15)
Rural area residence	4.73 (3.26 to 6.22)	−0.03 (−1.29 to 1.23)	−2.89 (−4.76 to −1.02)
Experiencing homelessness	−2.74 (−3.25 to −2.23)	−1.88 (−2.33 to −1.44)	−1.86 (−2.73 to −0.99)
Speaking language English	−1.07 (−2.56 to 0.41)	−1.27 (−3.18 to 0.63)	0.18 (−2.37 to 2.73)
Modified QCCI score	0.37 (0.29 to 0.47)	0.74 (0.67 to 0.82)	0.59 (0.46 to 0.72)
Federal poverty level, %	0.00 (−0.00 to 0.01)	−0.00 (−0.01 to 0.00)	0.00 (−0.01 to 0.01)

Abbreviations: AUD, alcohol use disorder; ED, emergency department; MH, mental health; QCCI, Quan-Charlson Comorbidity Index; SUD, substance use disorder.

^a^ Marginal effect reflects the change in the probability of receiving primary care follow-up attributable to a unit change in the explanatory variable. Primary care follow-up is defined as a visit with a primary care provider for an SUD or MH condition within 30 days of the qualifying ED visit.

^b^ ED claims for MH conditions with 30-d primary care follow-up for MH conditions.

^c^ ED claims for SUDs with 30-d primary care follow-up for SUDs.

^d^ ED claims for AUD with 30-d primary care follow-up for AUD.

^e^ Due to small sample sizes, Alaska Native and American Indian were initially separate racial identity categories that were consolidated into 1 category, as were Asian and Pacific Islander. Other race included those who identified as Hawaiian or other race at the time of enrollment.

### Thirty-Day Condition-Concordant Primary Care Follow-Up After SUD-Related ED Visits

Among Medicaid beneficiaries with ED visits for SUDs, characteristics associated with higher marginal probability of receiving 30-day condition-concordant primary care follow-up included older age (estimated ME, 0.05 [95% CI, 0.04-0.07] percentage points); Alaska Native or American Indian (estimated ME, 3.51 [95% CI, 2.68-4.33] percentage points), Asian or Pacific Islander (estimated ME, 3.06 [95% CI, 1.79-4.33] percentage points), non-Hispanic White (estimated ME, 4.70 [95% CI, 4.12-5.27] percentage points), or other race (estimated ME, 3.47 [95% CI, 2.21-4.71] percentage points) (referent, non-Hispanic Black); and higher modified QCCI score (estimated ME, 0.74 [95% CI, 0.67-0.82] percentage points). The only characteristic associated with lower marginal probability of receiving 30-day condition-concordant primary care follow-up after an ED visit for MH conditions was experiencing homelessness (estimated ME, −1.88 [95% CI, −2.33 to −1.44] percentage points; referent, not experiencing homelessness) (Table 2).

### Thirty-Day Condition-Concordant Primary Care Follow-Up After AUD-Related ED Visits

Among Medicaid beneficiaries with ED visits for AUD, characteristics associated with higher marginal probability of receiving 30-day condition-concordant primary care follow-up included older age (estimated ME, 0.04 [95% CI, 0.01-0.07] percentage points); female sex (estimated ME, 0.96 [95% CI, 0.21-1.70] percentage points; referent, male sex); Alaska Native or American Indian (estimated ME, 2.51 [95% CI, 1.05-3.96] percentage points), Asian or Pacific Islander (estimated ME, 2.47 [95% CI, 0.08-4.86] percentage points), non-Hispanic White (ME, 4.00 [95% CI, 2.87-5.13] percentage points), or other race (estimated ME, 3.07 [95% CI, 0.72-5.42] percentage points) (referent, non-Hispanic Black); and higher modified QCCI score (estimated ME, 0.59 [95% CI, 0.46-0.72] percentage points). Characteristics associated with lower marginal probability of receiving 30-day condition-concordant primary care follow-up after an ED visit for AUD included rural residence (estimated ME, −2.89 [95% CI, −4.76 to −1.02] percentage points; referent, urban) and experiencing homelessness (estimated ME, −1.86 [95% CI, −2.73 to −0.99] percentage points; referent, not experiencing homelessness) (Table 2).

## Discussion

In this statewide analysis of Medicaid claims in Washington, fewer than 15% of individuals received 30-day condition-concordant primary care follow-up after an ED visit for an MH condition, any SUD, or AUD. Because patients experiencing MH conditions, SUDs, and AUD can benefit from timely ongoing primary care engagement, low observed primary care follow-up rates are particularly concerning and suggest potential gaps in care coordination and primary care continuity.

It is important to recognize, however, that primary care is not the only potential venue for follow-up. Individuals with these conditions may instead engage with specialty behavioral health clinicians, residential programs, peer recovery support, or medication-assisted treatment programs not billed under primary care, which our study did not capture.

Notably, beneficiaries with ED visits for SUDs, including AUD, were less likely to receive 30-day condition-concordant primary care follow-up compared with those with MH conditions, highlighting potential care barriers such as stigma, limited care coordination, and reduced primary care access.^22,23^ Systems leaders and policymakers should consider adopting SUD- and AUD-specific primary care follow-up strategies after related ED visits for individuals not requiring intensive inpatient or outpatient management of their conditions. For example, EDs could design interventions initiating medication-assisted treatment with warm handoffs to primary care clinicians.^24,25,26^ Additionally, Medicaid and other payers could tie financial incentives to performance on condition-concordant follow-up for SUDs or AUD by using measures similar to the Follow-Up After Emergency Department Visit for Substance Use maintained by the National Committee on Quality Assurance or other accountability mechanisms.^27^

Racial differences in follow-up rates were evident across our study populations. Non-Hispanic Black individuals and those experiencing homelessness had consistently lower probabilities of receiving follow-up compared with other groups, raising concern for potential differences in access to care. Amid well-documented access barriers facing these populations,^28,29,30^ our findings suggest the need for tailored care coordination and outreach strategies, such as care navigation or primary care street medicine follow-up, to improve continuity of and access to primary care services among populations experiencing MH conditions or all SUDs.^31^ An additional finding among AUD-related ED visits in our subgroup analysis (ie, association between rural residence and lower probability of receiving condition-concordant follow-up) also raises the possibility of geographic differences creating unique access barriers related to AUD. Lower rates of follow-up and treatment among rural beneficiaries are consistent with studies examining other medical conditions.^32,33^ Geographic availability of care may help contextualize findings specific to AUD. Prior work has shown that AUD treatment resources are disproportionately concentrated in urban areas, which may limit follow-up options for rural beneficiaries, highlighting the potential role of strategies such as telehealth-enabled care and stronger referral pathways to improve continuity of AUD care in rural settings.^34^ Further work is needed to better elucidate underlying drivers and potential solutions for these observed differences, such as enhanced telehealth services for rural populations.^35,36^

In contrast with findings related to social and demographic factors, our study found that higher modified QCCI scores were consistently associated with a modest but statistically significant increase in the probability of receiving 30-day condition-concordant primary care follow-up across MH condition–, SUD-, and AUD-related ED visits. This finding suggests that individuals with conditions of greater overall medical complexity may be more likely to receive timely primary care follow-up, potentially reflecting better established care coordination and follow-up processes for patients with multiple physical comorbidities. It is also possible that individuals with greater physical comorbidity may already have an established primary care clinician, allowing for expedient follow-up. Identifying whether primary care continuity preceding an MH condition–, SUD-, or AUD-related ED visit facilitates greater probability of receiving timely condition-concordant follow-up could be an area of future study.

Additionally, age- and sex-related differences in follow-up may reflect condition-specific barriers and social perceptions. Our findings suggest that older beneficiaries may face stigma or lower prioritization for MH follow-up, while older individuals with SUDs or AUD may have more established treatment pathways or comorbidities prompting primary care engagement. Similarly, more frequent follow-up among female beneficiaries for MH conditions and AUD may reflect differences in care engagement, highlighting the need to consider how demographic factors intersect with condition-specific care patterns.

Overall, our study highlights broader primary care access challenges. In 2025, approximately 92 million US residents lived in designated primary care health profession shortage areas, nearly two-thirds of which were rural.^37^ Additionally, in the decade preceding the study period, a 21% relative increase was observed in the percentage of US adults without a usual source of care, thus limiting access to primary care follow-up after ED visits.^38^ These challenges are also reflected in Washington State, where all 69 counties are considered shortage areas and 23% of adults lack a usual source of care.^39,40,41^ Collectively, these broader primary care access issues may contribute to less availability of primary follow-up services after MH condition–, SUD-, and AUD-related ED visits. Therefore, to ensure access to these services, not only must primary care workflows change to prioritize follow-up for these conditions, but investments must also be made to bolster primary care infrastructure in general.

Our study’s focus on Medicaid beneficiaries is also notable in the context of the One Big Beautiful Bill Act. This act is expected to increase the number of uninsured individuals by nearly 11 million nationally and by 450 000 in Washington State, predominantly via loss of Medicaid enrollment.^42,43^ Prior studies have demonstrated that uninsured individuals are less likely to receive timely primary care follow-up after ED visits or hospitalizations than individuals with commercial or Medicaid insurance.^44,45^ Therefore, the loss of insurance coverage may exacerbate already low primary care follow-up rates for MH conditions, SUDs, and AUD, thereby increasing reliance on ED care among uninsured individuals.

### Limitations

There were limitations to this study. First, as a single-state analysis, findings may not be generalizable to other states, particularly considering variability in how Medicaid programs are administered. Second, primary care clinicians may have addressed an MH condition or SUD during an ED follow-up visit without using the diagnosis code in the claim, which may have led to underestimation of condition-concordant follow-up. Third, as a cross-sectional analysis of 2022 data, findings may not be generalizable to other years during which COVID-19–related ED and primary care disruptions may have varied. Fourth, we examined 30-day condition-concordant primary care follow-up after all ED visits for the conditions of interest, regardless of ED discharge disposition (eg, subsequent hospitalization or leaving the ED against medical advice). Because our data lacked hospital discharge dates, follow-up was measured from the index ED visit for all beneficiaries. As a result, we could not distinguish differences in follow-up timing between beneficiaries discharged home and those hospitalized after the ED visit. Fifth, our analysis focused on condition-concordant primary care follow-up and did not capture follow-up occurring in other settings, such as specialty behavioral health clinics, addiction medicine programs, residential treatment facilities, and detoxification centers. This limitation is particularly relevant for individuals with SUDs and AUD, for whom follow-up care may preferentially occur outside of primary care. We did not examine these alternative follow-up modalities, because claims-based identification of specialty behavioral health and addiction treatment is heterogeneous and often lacks the consistent diagnosis coding necessary to reliably assess condition concordance. Therefore, our findings should be interpreted as reflecting gaps in primary care follow-up specifically, rather than as an absence of any follow-up care.

## Conclusions

In this cohort study of Medicaid beneficiaries in Washington who visited the ED, the rate of condition-concordant primary care follow-up after ED visits for MH conditions, all SUDs, and AUD was low. Additional policy or workflow adjustments may be necessary to facilitate beneficial primary care follow-up after ED use, particularly in view of observed differences in follow-up after SUD- compared with MH condition–related ED visits and observed racial and socioeconomic differences. Such adjustments could include tailored care coordination and outreach strategies to improve continuity and access to primary care among populations experiencing MH conditions or SUDs.

## Notes

Supplement 1. **eMethods 1.** ICD-10 Code Diagnoses Included as Mental Health Conditions in Identifying ED and Primary Care Visits **eMethods 2.** ICD-10 Code Diagnoses included as Substance Use Disorders in Identifying ED and Primary Care Visits **eMethods 3.** Current Procedural Technology Codes Used to Identify Primary Care Follow-Up Visits

Supplement 2.
Data Sharing Statement
