# Error in the Abstract

**DOI:** 10.1001/jamanetworkopen.2026.16454

**Published:** 2026-05-08

In the Original Investigation titled “Primary Care Follow-Up After Mental Health and Substance Use Emergency Department Visits in Medicaid,”^1^ which was published on April 14, 2026, there was an error in 95% CI for alcohol use disorder in the last sentence of the Results section in the Abstract. The data should appear as follows: −1.86 [95% CI, −2.73 to −0.99] percentage points. The article has been corrected.
