# Supplementary material for: Primary Care Follow-Up After Mental Health and Substance Use Emergency Department Visits in Medicaid
Source: JAMA Netw Open. 2026 Apr 14;9(4):e264917. doi: 10.1001/jamanetworkopen.2026.4917 (PMC13080537; doi:10.1001/jamanetworkopen.2026.4917)
Supplement: Supplement 1. — eMethods 1. ICD-10 Code Diagnoses Included as Mental Health Conditions in Identifying ED and Primary Care Visits eMethods 2. ICD-10 Code Diagnoses included as Substance Use Disorders in Identifying ED and Primary Care Visits eMethods 3. Current Procedural Technology Codes Used to Identify Primary Care Follow-Up Visits [file jamanetwopen-e264917-s001.pdf]

Supplemental Online Content

Staloff JA, Wong ES, Joo JH, et al. Primary care follow-up after mental health and substance use emergency department visits in Medicaid. *JAMA Netw Open*. 2025;9(4):e264917. doi:10.1001/jamanetworkopen.2026.4917

eMethods 1. ICD-10 Code Diagnoses Included as Mental Health Conditions in Identifying ED and Primary Care Visits

eMethods 2. ICD-10 Code Diagnoses included as Substance Use Disorders in Identifying ED and Primary Care Visits

eMethods 3. Current Procedural Technology Codes Used to Identify Primary Care Follow-Up Visits

This supplemental material has been provided by the authors to give readers additional information about their work.

© 2026 Staloff JA et al. *JAMA Network Open*.

eMethods 1. ICD-10 Code Diagnoses included as Mental Health Conditions in Identifying ED and Primary Care Visits

Schizophrenia, schizotypal, delusional, other

Mood (affective) disorders

Anxiety, dissociative, stress-related, somatoform, other nonpsychotic mental disorders

Behavioral syndromes associated with physiological disturbances and physical factors

Disorders of adult personality and behavior

Intellectual disabilities

Pervasive and specific developmental disorders

Behavioral, emotional disorders, unspecified disorders

© 2026 Staloff JA et al. *JAMA Network Open*.

eMethods 2. ICD-10 Code Diagnoses included as Substance Use Disorders in Identifying ED and Primary Care Visits

Alcohol-related disorders

Opioid-related disorders

Cannabis disorders

Sedative disorders

Stimulant disorders

Hallucinogen disorders

Inhalant disorders

Other specified substance-related disorders

© 2026 Staloff JA et al. *JAMA Network Open*.

eMethods 3. Current Procedural Technology Codes Used to Identify Primary Care Follow-Up Visits

Supplement 3.

Telehealth Visits

CPT codes: 99201, 99202, 99203, 99204, 99205, 99211, 99212, 99213, 99214, 99215, 99241, 99242, 99243, 99244, 99245, 99441, 99442, 99443

Modifiers: 95, GT, GQ, CR G0

In-Person Visits

CPT codes: 99201, 99202, 99203, 99204, 99205, 99211, 99212, 99213, 99214, 99215, 99241, 99242, 99243, 99244, 99245, 99381, 99382, 99383, 99384, 99385, 99386, 99387, 99391, 99392, 99393, 99394, 99395, 99396, 99397, G0438, G0439

© 2026 Staloff JA et al. *JAMA Network Open*.
