# Supplementary material for: Primary Care Follow-Up After Mental Health and Substance Use Emergency Department Visits in Medicaid
Source: JAMA Netw Open. 2026 Apr 14;9(4):e264917. doi: 10.1001/jamanetworkopen.2026.4917 (PMC13080537; doi:10.1001/jamanetworkopen.2026.4917)
Supplement: Supplement 2. — Data Sharing Statement [file jamanetwopen-e264917-s002.pdf]

Data Sharing Statement

Staloff. Primary Care Follow-Up After Mental Health and Substance Use Emergency Department Visits in Medicaid. *JAMA Netw Open*. Published April 14, 2026. doi:10.1001/jamanetworkopen.2026.4917

Data
Data available: No

Additional Information
Explanation for why data not available: The data will not be shared as the authors do not own the rights to the data. The data are owned by Washington State Medicaid and the authors were able to use the data via a data use agreement specific to the University of Washington.
